# Spatial distribution of gamma radiation dose rates from natural radionuclides and its radiological hazards in sediments along river Iju, Ogun state Nigeria

**DOI:** 10.1016/j.mex.2020.101086

**Published:** 2020-10-07

**Authors:** Omeje Maxwell, Adewoyin Olusegun O., Joel Emmanuel S., Ikechukwu Ijeh B., Omeje Uchechukwu A., Ayanbisi Oluwasegun, Iyanuoluwa Ogunrinola E., Timothy Terhile Angbiandoo M., Oha Ifeany A., Mohammad Alam Saeed

**Affiliations:** a Department of Physics, College of Science and Technology, Covenant University, P.M.B 1023, Ota, Ogun State, Nigeria; b Department of Physics, Michael Okpara University of Agriculture, Umudike, Umuahia, Abia State; c Department of Public and Community Health Care, College of Medicine, Idiaraba, University of Lagos, Nigeria; d Department of Geology, Faculty of Science, University of Nigeria, Nsukka, Nigeria; e Division of Science and Technology, University of Education Township Lahore-Pakistan, Pakistan

**Keywords:** Soil sediment, Iju River, Gamma spectroscopy, Radioactivity, Radiological parameters, Excess lifetime cancer risks, Multivariat analysis

## Abstract

This study assessed the radioactivity level of Iju River sediments and its associated radiological risks exposure to human. Gamma absorbed dose rates were measured using a portable gamma spectrometer at an interval of 50 m between each point (Model No:RS-125 manufactured by Canadian Geophysical Institute). The measured absorbed gamma dose rates range from 22 to 59 nGy/h with the highest value noted in site 13. The mean activity concentrations of U-238, Th-232 and K-40 from Iju river sediments were 24.1 ± 0.4 Bq/kg, 35.2 ± 1.1 Bq/kg and 501.0 ± 11.1 Bq/kg, which were lower than the world average values of 32 and 45 for U-238 and Th-232 by 25% and 21% and higher than 412 Bq/kg for K-40 by a factor of 0.22 (22%).

• This highest value of K-40 found in site 6 may be due to the presence of feldspartic minerals buried in the sediments.
• Consequently, the radiological hazard indices were below the permissible limits except the mean value of 28 × 10^−2^ for excess lifetime cancer risk (ELCR) for the samples collected in site 6, which is higher than the average world value of 0.29 × 10^−3^ by a factor of 0.03.
• The regression model indicates that Th-232 attributes more radioactivity impacts on the soil sediments than the contributions of U-232 and K-40, as such, presumed to be the radionuclide parameter controlling the depositional process in the region.

## Highlights

• Radioactivity in soil sediments of Iju River was found to be within the limit level.
• Excess lifetime cancer risks on human and ecosystem found to be higher slightly in some sites.
• Activity utilization index from gamma radiation of natural radionuclides found to be within the permissible level.

## Graphical absrtact

The Graphical Abstract of the Radionuclides Exposure to the Public living in Different Communities along Iju River

**Figure:**
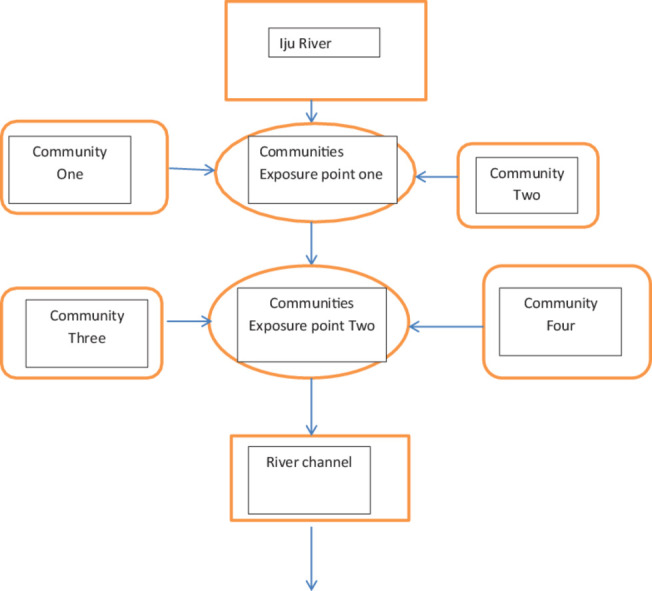

Specification table

**Table:** 

Subject area	Environmental Sciences
More specific subject area	Environmental Radioactivity
Method name	Gamma spectroscopy analysis
Name and reference of original method	R. Ravisankar, S. Sivakumar, A. Chandrasekaran, J. Prince Prakash Jebakumar, I. Vijayalashmi, P. Vijayagopal, B. Vaenkatraman (2014) Spatial distribution of gamma radioactivity levels and radiological hazard indices in the East Coastal sediments of Tamilnadu, India with statistical approach, Radiation Physics and Chemistry; 103, 89 −98 https://doi.org/10.1016/j.radphyschem.2014.05.037 Mama C. N., C. C. Nnaji, P. C. Emenike, C. V. Chibueze, (2020).Potential environmental and human health risk of soil and roadside dust in a rapidly growing urban settlement, International Journal of Environmental Science and Technology https://doi.org/10.1007/s13762–020–02637–9 Omeje M, Wagiran H, Ibrahim N, Lee SK, Soheil, S (2013a) Comparison of ^238^U, ^232^Th, and ^40^K in different layers of subsurface structuresion Dei-Dei and Kubwa, Abuja. Radiation Physics and Chemistry,91, 70 – 80 https://doi.org/10.1016/j.radphyschem.2013.05.006
Resource availability	The resources for reproduction of the same methods such as software, samples and other materials necessary are described in details could be found in the Methods section

## Method overview

Naturally occurring radionuclides can be transferred from soil to plants, animal, and then exposed to human [1]. Radionuclides could appear as toxic elements and undergo bioaccumulation and bioconcentration, resulting in adverse impact to human and its environs [2]. Evaluation of radioactivity in soil and sediments in an environment is useful for the protection of human health and harmful effects which is of great interest [3]. These measured radioactive sources in the marine environment could be from either natural particles of the Earth's crust manifesting in all terrestrial ecosystem or from the anthropogenic source [3,4]. Another radioactivity measurements indicate that the threat to the marine environment is the anthropogenic sources such as industrial activities and mining [4]. Even though the West African coastal region lacks information about the nuclear industry operations, mining, industrialization, agricultural production, offshore gas and oil exploration could be attributable to the radioactivity level of an environment [5]. The measured radiation exposure from soil to human may either originate from the primordial radionuclides or the external radiation present in soil [6]. The radiation from radon and its decay products that sometimes emanate from the soil sediments internally affects the human respiratory tracks [1].

The activity concentrations of radionuclides in the natural ecosystem has resulted in an understanding of the health implications over the past years. These radionuclides penetrate the tissues of the marine species through different mechanisms, hence, penetrating the food chain through the ingestion of sea or marine food [7].

Conversely, to improve and sustain river health system, an accurate assessment of the current radioactivity and its radiological exposure to the ecosystem is highly needed [8]. Researchers have mapped out a holistic approach to quantify and evaluate river water quality parameters and their risk exposure to the human and environmental ecosystem [8].

This study is aimed at assessing the radioactivity level of Iju river sediment and its radiological hazards to the inhabitants of the Environment. Moreso, to set a baseline of the radiological parameter of Iju river that connects to other rivers in Southwest Nigeria.

## Study area

**Fig. 1:**
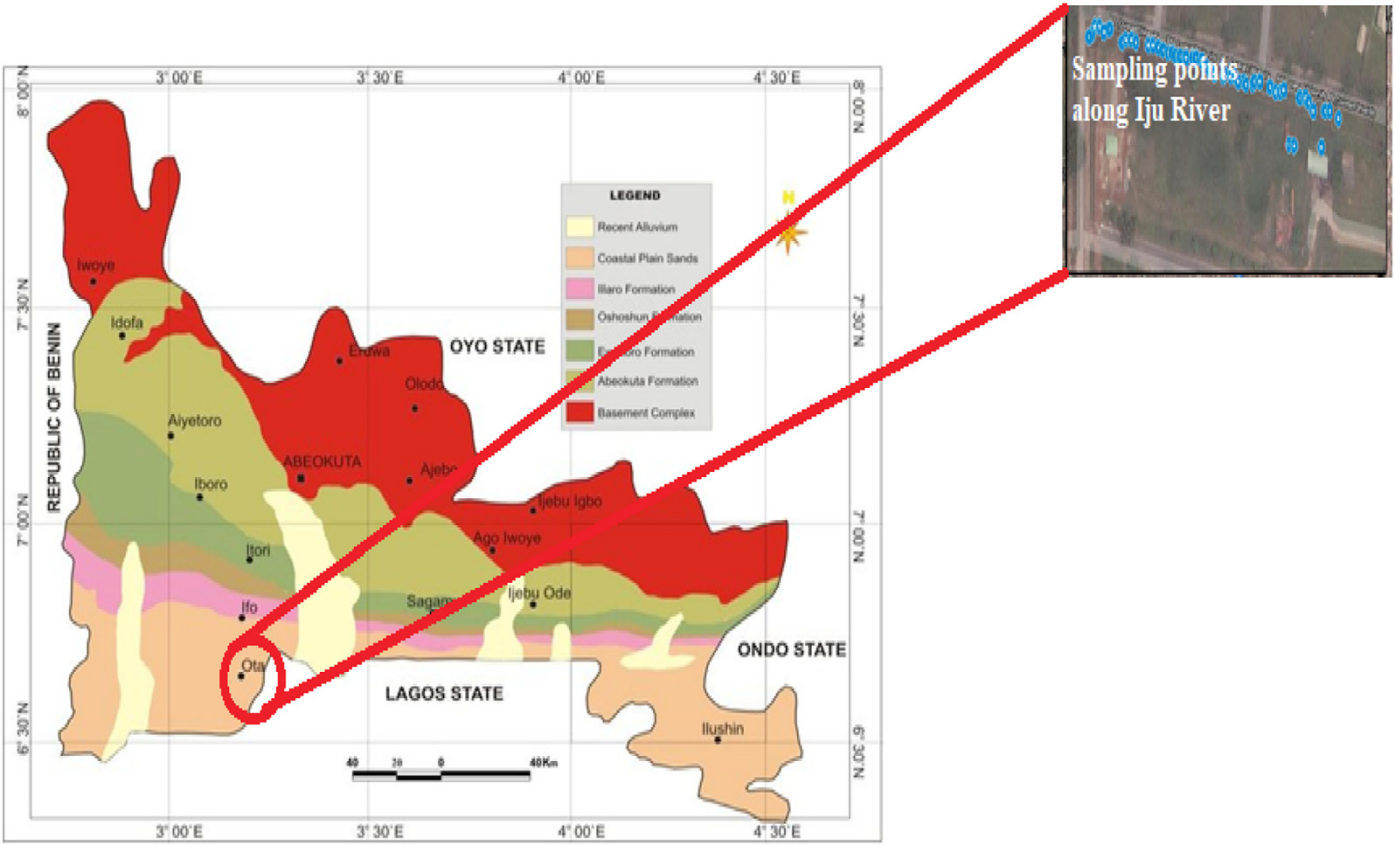
Geological map of ogun state with red circle showing the study area [10].

The study area is located in Ogun State, South-West, Nigeria, which lies within the latitude 6.680851°N and longitude 3.148471°E. Ogun State is an inland state and is bounded to the North by Oyo and Osun States, to the South by Lagos State, to the East by Ondo State and to the West by the Benin Republic. Ogun State has a tropical climate with an average temperature and rainfall of 27.1 °C and 1238 mm respectively. Ogun State with other south-western Nigerian states including Oyo and Lagos States, lies in the eastern Dahomey Basin. Its geology is composed of sedimentary and basement complex rocks. The sedimentary rocks are Late Cretaceous to Early Tertiary in age [9]. Stratigraphically, the sedimentary rock of Ogun State consists of the Abeokuta group, Imo group, Ewekoro, Oshosun, Ilaro and Benin Formations [9]. The Cretaceous Abeokuta group constitutes the Ise, Afowo and Araromi Formations and lies on top of the basement complex. The Abeokuta Group is being overlain by the Ewekoro, Oshosun and Ilaro Formations which are all overlain by the Benin Formation made up of coastal plain sands [9] is shown in Fig. 1

### The coastline sediments of Iju River

The coastal nature of the Iju river sediments shows some parts where communities have access to the river for fishing and fetching water for domestic purposes. They are almost parallel to the coastline sediments of other rivers such as River Atuara which is located about 5 km away from Iju town. The deposits of river Iju comprise of mudflats, salt marsh and inner sandy flats. Within the river sub-environments, it cuts across the creeks and bordering areas. The sub-environments along the coastal river is characterized by surface features such as vegetation, an association of different sediments, sedimentary structures and textures. The sediments contain high contents of iron, phosphate, nitrate and sulphates [10]. The tidal water along the river decreases its capacity towards the intertidal zone, which increases the sediments deposits and as well reduces the size of the grains. These processes seem to be modified by the secondary agents caused by waves for rearrangements of the sediments in the study area.

## Measurements of gamma radiation dose level

Portable hand-held radiation detector (Super-SPEC RS-125) from Canadian Geophysical Inc. was used to measure the background gamma dose level in the study area. This instrument is most suitable for detecting naturally occurring radionuclides and dose exposure. The equipment has a high degree of accuracy with probable measurement errors of about ±5%. The portable equipment has an incorporated design and direct assay read-out values, storage data point with weather protection, easy to use and highly sensitive. The number of count display of RS-125 Super-SPEC in the front side of the panel in cps at 1/*sec* update rate. The variable rate counts of the SCAN mode of RS-125 Super SPEC usually stores data in the memory of the device through Bluetooth connection to external storage of the hand-held device. The location of the data is gotten through the connection of External Global Positioning System (GPS) to the data stream via Bluetooth connection to the device. In the study area, the measurements were taken at intervals of 50 m intervals following the regions with peak sediments deposit, gully and weathered surface areas. Few zones of lower sediments from the river banks were taken as control. At each station, 4 different measurements were taken and the average obtained was used to represent the actual data point for that site. At each point of measurements, the sediment sample was collected for laboratory gamma ray spectroscopy counting. The background measurement is provided by the assay mode of RS-125 Super SPEC and dose rate data is directly acquired in nGy/h. The RS-125 Super SPEC comes with utility software which is used to download the statistics record that is stored in memory and further connected to the computer through Bluetooth or USB. The measured data stored in excel sheet with proper coordinates was processed, georeferenced and interpolated using ArcGIS (version 10.8) spatial analyst.

### Method of GIS analysis of background dose rates data samples measured along Iju River

The spatial distribution of gamma dose rates in sediments of Iju River was first carried out using an interpolated scheme with the inverse distance weighing interpolation function being applied on all the sediment samples measured. The interpolated functions were used as input to the ArcGIS 10.8. [11,12]

### Sample collection and preparation

In the study area, a total number of 13 sediment samples along Iju River were scooped from a depth of 10 cm to collect 1000 g of samples at each marked site within a distance of 50 m from one another. These points were chosen based on the areas that communities access the river water for consumption and other domestic purposes. Each sediment sample was air-dried under the ambient temperature of 29 °C for one week [13]. The soil sediment samples were crushed, powdered to a maximum grain size of 1 mm, dried in an oven at approximately 105 °C until the samples maintained a constant weight of about 500 g. Each sample was sealed in high-density polyethene plastic bottles, labelled accordingly and sent to Activation analysis Laboratory in Canada for gamma counting. All the samples were sealed in a radon impermeable plastic container for 4 weeks to bring Rn-222 and its short-lived radionuclide daughters products into equilibrium with Ra-226 [12,13].

### Method for gamma spectroscopy analysis of sediment samples

The concentrations of U-238, Th-232 and K-40 measured in Bqkg^−1^ dry weight of the samples from Iju River were analysed using gamma-ray spectrometry method. A NaI (TI) detector 3′x 3′was used with proper lead shielding to reduce the background contribution by a factor of about 95%. The determination of various radionuclides concentrations of interests in Bqkg^−1^ was measured using count spectra. The photo peaks (region of interest) of the gamma-ray corresponds to 1.46 MeV for K-40, 1.76 MeV for Bi-214 and 2.614 MeV for TI-208 considered to be the activities of K-40, U-238 and Th-232, respectively in the Iju River soil sediment samples. The NaI(TI) detector used has detection limit of 8.50, 2.21 and 2.11 Bqkg^−1^ for K-40, U-238 and Th-232, respectively [13]. The counting time of 21000s for each sample was adopted [12]. The activity concentrations were calculated using Eq. (1) [14].

(1) $$C_{s}=C_{ref}\frac{\frac{P_{s}}{D_{s}}-\frac{P_{b}}{D_{b}}}{\left(\frac{P_{ref}}{D_{ref}}-\frac{P_{b}}{D_{b}}\right)\;M_{s}}$$

Where Cs and Cref are the activity concentrations in Bq/kg of the measured sediment samples and standard reference materials, respectively. P_s_, P_ref_ and P_b_ are the photopeak areas of the sediment samples, standard reference materials and the background photopeak gamma limes, respectively, which is dimensionless. Also, D_s_, D_ref_ and D_b_ are the counting duration/time in seconds for the sediment samples, standard reference materials, and background, respectively. The uncertainty of the activity level from the samples was determined using Eq. (2) [14].

(2) $$Q=C_{s}\sqrt{\left({\left(Q_{C_{ref}}\right)}^{2}+\frac{{\left(Q_{s}\right)}^{2\;}+{\left(Q_{b}\right)}^{2\;}}{{\left(R_{s}-R_{b}\right)}^{2\;}}+\frac{{\left(Q_{ref}\right)}^{2\;}+{\left(Q_{b}\right)}^{2\;}}{{\left(R_{ref}-R_{b}\right)}^{2\;}}\right)}$$

Where Q is the uncertainty of the samples, Q_Cref_ is the relative uncertainty of the reference materials; Q_ref_, Q_s_ and Q_b_ are the rate count uncertainty of the standard reference materials, sediment sample, and background, respectively. R_ref_, R_s_ and R_b_ are the net count of gamma line energies of the radionuclides in the sediment samples, standard reference materials and background, respectively.

### Multivariate analysis

Initial statistical two way ANOVA was run on Excel to compare the variation in the sites with the observed days. The regression analysis was performed in R-Studio 3.0.2 version. The categorical variables were converted into dummy variables in Excel such that the numbers “0” and “1” can be used to identify each value of the variables accordingly. Thereafter, the dummy variables were fed into R-Studio 3.0.2 version alongside the dependent variables. The results of the analysis were generated in the command window of the software.

## Results and discussion

### Spatial distribution of background radiation dose rate from the sediment samples in the study area

**Fig. 2:**
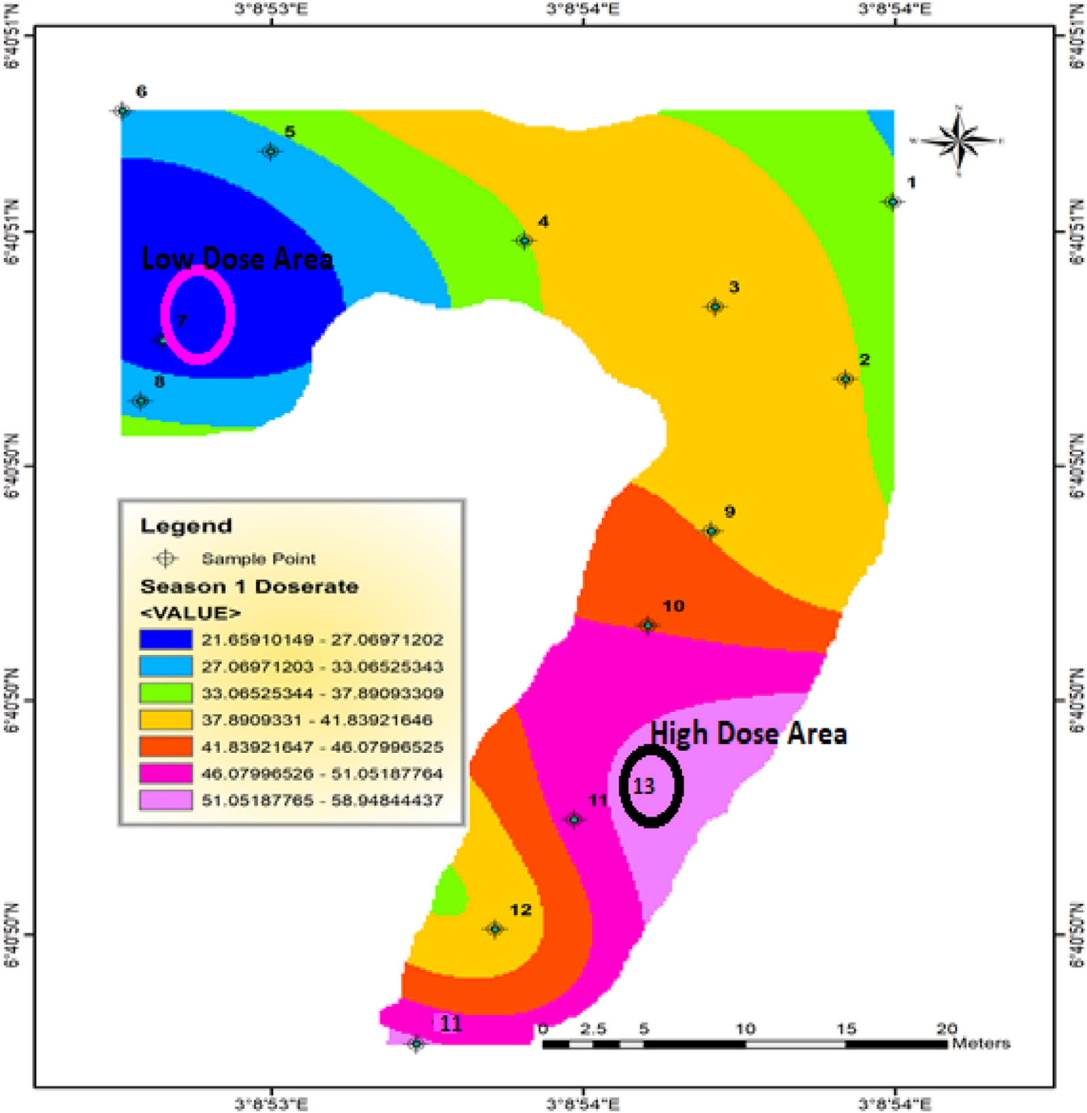
Spatial distribution of background dose rates from the sediment samples of Iju River circled the high and low background zones.

Fig. 2 presents the result of the ArcGis spatial distribution of dose rates measured along the River Iju using the data presented in Table 1. It can be observed from Fig. 2 that the background distributions of gamma dose rates comprises of different radiation highs and lows in the area. The Iso-dose column with the highest background gamma dose was identified in site 13 with backgeound dose rates ranging from 21.6 to 58.9 nGy/h . The corresponding mean value is 37.1 nGy/h, which is distinctly lower than the world average value of 59 nGy/h suggested by [16] by a factor of 0.37 (approximately 37%). This shows that the zone may not pose higher radiation risks when compared to the international reference level by UNSCEAR, [16]

**Table 1:** The gamma dose rate measured in the Study area.

Location	Gamma Dose rate (nGy/h)
ST1	34.2
ST2	38.4
ST3	21.6
ST4	37.8
ST5	31.2
ST6	30.8
ST7	24.1
ST8	30.9
ST9	41.6
ST10	45.9
ST11	47.9
ST 12	39.1
ST13	58.9
Mean values	37.1
(UNSCEAR, 2000)	59

### Activity concentrations of U-238, Th-232 and K-40 from Iju River soil sediment samples

The activity concentrations of U-238, Th-232 and K-40 emitted from the soil sediment samples are presented in Table 2. The activity concentrations of U-238, Th-232 and K-40 range from 14.5 ± 0.1 to 31.8 ± 1.2 Bq/Kg, 22.6 ± 0.3 to 48.0 ± 1.9 Bq/Kg and 148.7 ± 5.3 to 852.9 ± 14.7 Bq/K, respectively, with the corresponding mean values of 24.1 ± 0.4 Bq/Kg, 35.2 ± 1.1 Bq/Kg and 501.0 ± 11.1 Bq/Kg, respectively. This study revealed distinctly non-uniform distribution of U-238, Th-232 and K-40 with the highest values of 31.8 ± 1.2 Bq/Kg, 48.0 ± 1.9 Bq/Kg and 852.9 ± 14.7 Bq/K were found in site 13, 11 and 2, respectively. These higher values may be due to the localized buried minerals assembled in the sediments presumed to be secondary deposition. The lowest values for U-238, Th-232 and K-40 were found in site 7,6 and 11, respectively. The mean values of 24.1 ± 0.4 Bq/kg and 35.2 ± 1.1 Bq/kg for U-238 and Th-232 obtained from this study were compared with the international recommended values of 33, 45 and 420 Bq/kg according to [16] were found to be lower by factors of 0.27 (approximately 27%) and 0.22 (approximately 22%), whereas the K-40 with a mean value of 501.0 ± 11.1 Bq/kg is higher by a factor of 0.19 (approximately 19%). The lower value of uranium may be due to its oxidative nature in aqueous phase whereas the higher value of K-40 may be attributed to the feldspathic minerals redeposited in site 2.

**Table 2:** Activity concentrations of U-238, Th-232 and K-40 from Iju River sediments.

Location	U-238 (Bq/Kg)	Th-232 (Bq/Kg)	K −40 (Bq/Kg)	Longitude (Dec. Deg.)	Latitude (Dec. Deg.)
ST1	23.4 ± 0.4	33.0 ± 0.8	398.4 ± 9.5	3.148471	6.680851
ST2	24.0 ± 0.6	36.7 ± 1.1	852.9 ± 14.7	3.14845	6.680746
ST3	19.1 ± 0.2	42.6 ± 1.5	426.3 ± 10.2	3.148392	6.680789
ST4	28.0 ± 0.8	34.9 ± 0.8	514.3 ± 11.0	3.148307	6.680828
ST5	29.0 ± 1.2	27.1 ± 0.5	331.3 ± 9.0	3.148194	6.680881
ST6	28.4 ± 0.9	22.6 ± 0.3	920.1 ± 15.6	3.148128	6.680905
ST7	14.5 ± 0.1	24.7 ± 0.3	379.2 ± 9.3	3.148146	6.680769
ST8	23.1 ± 0.4	23.8 ± 0.3	458.8 ± 10.6	3.148136	6.680733
ST9	24.3 ± 0.5	39.8 ± 1.2	652.7 ± 12.8	3.14839	6.680656
ST10	25.0 ± 0.6	39.5 ± 1.2	203.4 ± 8.1	3.148362	6.6806
ST11	21.6 ± 0.3	48.0 ± 1.9	148.7 ± 5.3	3.148329	6.680485
ST 12	20.0 ± 0.3	39.5 ± 1.2	728.2 ± 13.4	3.148294	6.68042
ST13	31.8 ± 1.2	45.4 ± 1.7	498.2 ± 10.9	3.148259	6.680352
Mean values	24.1 ± 0.4	35.2 ± 1.1	501.0 ± 11.1		
(Radiation, 2000)	33	45	420		

Some individual values for Th-232 and K-40 activity levels for each sampling point as well as the overall mean for K-40 were higher when compared to the world average values. Significantly, K-40 which is far higher than the world average was scooped from the soil in site 6 with a value of 920.1 ± 15.6 Bq/kg which is the channel of the river-laterite contact zone.

### Radiological health risk assessment

The United Nations Scientific Committee on the Effects of Atomic Radiation (UNSCEAR) and the International Atomic Energy Agency (IAEA) has identified a number of risks that can be taken into account when examining radiation exposure to human in the environment.

#### Radium equivalent (R_equ_)

In order to assess the radiation hazard associated with the soil sediments samples as presented in Table 3, the Ra_equ_ is a weighted sum of the radioactivity level of Ra-226, Th-232 and K-40 in the soil sediment sample. This allows the comparison with their individual Ra-226, Th-232 and K-40 activity concentrations [15]. This is assumed that all Ra-226 and Th-232 decay products are in radioactive equilibrium with their progeny. This Radium equivalent will provide a guideline in regulating the general public safety standard of radioprotection of the people residing in the area. This index is the most widely used to assess the radiation hazard which is calculated according to Eq. (3) [14,16]

(3) $$Ra_{eq}=C_{U}+1.43C_{Th}+0.077C_{K}$$

where C_U_, C_Th_ and C_K_ are the specific activities of U-238, Th-232 and K-40 measured in Bq/kg respectively. This formula estimates that 1 Bq/kg of U-238, 0.7 Bq/kg of Th-232 and 13 Bq/kg of K-40 produces the same gamma dose rates. The Ra_equ_ is related to both internal doses due to the radon and external gamma dose and should have the highest value of 370 Bq/kg [15,16]. It can be observed from Table 3 that the Ra_equ_ varies between 79.1 and 233.7 Bq/kg with the highest value found in site 13 sample. The lowest value of 79.1 Bq/kg Ra_equ_ activities was noted in site 7. Comparing the mean values of 113.0 and 144.0 Bq/kg, respectively, for Ra_equ_ activity from the two different days with the international standard value of 370 Bq/kg according to [15], the highest value (144.0 Bq/kg) for this present study is more than twice lower. These lower values indicate that the samples may not pose any radiological risks to the public in the area.

**Table 3:** Radium equivalent (R_equ_) and absorbed dose rate (DC) from River Iju sediments samples.

Location	R_eus_ (Bq/Kg)	DC (nGy/h)
ST1	101.5	47.4
ST2	142.3	68.9
ST3	112.9	52.4
ST4	117.6	55.5
ST5	93.3	43.6
ST6	131.6	65.2
ST7	79.1	37.5
ST8	92.6	44.2
ST9	131.7	62.6
ST10	97.3	43.9
ST11	101.7	45.2
ST 12	132.8	63.6
ST13	135.2	62.9
Mean value	113.0	53.3
World Average Values	370	84

#### Absorbed dose rate (DC)

The dose rate is the outdoor absorbed dose rate in nGy/h in the air from terrestrial gamma radiation at 1 m above the ground which is calculated by using nGy/h per Bq/kg conversion factor to transform the specific activities of C_Ra_, C_Th_ and C_K_ in the sediment samples into absorbed dose rate [16,17]. Eq. (4) is used to calculate the absorbed dose rates due to the radionuclides gamma radiation in the air.

(4) $$D_{c}=0.462\;C\left(U-238\right)+0.604\;C\left(Th-232\right)+0.0417\;C\left(K-40\right)$$

From Table 3, the minimum gamma dose rate was found in site 7 with a value of 37.5 nGy/h, while the maximum value of 68.9 nGy/h reported in site 2. The estimated mean value found in Iju river sediment samples is 53.3 nGy/h, and about 0.37 factor (37%) lowe than the with the world average value of 84 nGy/h of [16]. The lower values of the absorbed gamma dose rates may be due to lower suspended sediments that fall to the stream bed to become bottom sediments.

#### Internal hazard index (H_int_)

The H_int_ index from the sediment samples is shown in Table 4. The internal exposure to radon and its progeny can be quantified using the internal index which is calculated using Eq. (5) [15,^16^,21]

(5) $$H_{int}=\left\{\frac{C_{U}}{185}+\frac{C_{Th}}{259}+\frac{C_{K}}{4810}\right\}\leq 1$$

**Table 4:** The internal hazard index (*H_int_*), external hazards index (*H_ext_*), annual effective dose (*AEDE*), gamma activity index (I_ɣ_), alpha index (Iα), activity utilization index (AUI) and excess lifetime cancer risk (ELCR) from Iju River sediment samples.

Location	H_int_	H_ext_	AEDR (mSv/y)	Gamma index	Alpha Index	AUI	ELCR
ST1	0.34	0.27	0.06	0.38	0.51	0.65	0.20
ST2	0.45	0.38	0.08	0.55	0.71	0.74	0.30
ST3	0.37	0.31	0.06	0.42	0.56	0.73	0.23
ST4	0.39	0.32	0.07	0.44	0.59	0.72	0.24
ST5	0.33	0.25	0.05	0.34	0.47	0.62	0.19
ST6	0.43	0.36	0.08	0.52	0.66	0.61	0.28
ST7	0.25	0.21	0.05	0.30	0.40	0.46	0.16
ST8	0.31	0.25	0.05	0.35	0.46	0.54	0.19
ST9	0.42	0.36	0.08	0.50	0.66	0.76	0.27
ST10	0.33	0.26	0.05	0.35	0.49	0.72	0.19
ST11	0.33	0.27	0.06	0.36	0.51	0.79	0.20
ST 12	0.41	0.36	0.08	0.51	0.66	0.72	0.27
ST13	0.45	0.36	0.08	0.50	0.68	0.89	0.27
Mean	0.37	0.31	0.07	0.42	0.57	0.69	0.23
World Average Values	1	1	0.07	2	1	2	0.29 × 10^−3^

For the utilization of sediment samples to be considered safe, the internal hazard must be less than 1 [18], [19], [20], [21]. In this present study, the H_int_ varies from 0.25 to 0.45 with a mean value of 0.37. The highest value of 0.45 was found in site 2 and 13, while the lowest value was noted in site 7. The mean value of 0.37, which is far less than the world average of < 1, indicating that the internal hazard index is lower than the critical. The average values are twice lower than the recommended safe level when compared to [16,^18^,21] and are considered safe for general public residing in the area

#### External hazard index (Hex)

The H_ext_ index obtained from U-238, Th-232 and K-40 gamma emission from the sediment samples are presented in Table 4. The purpose of this risks index is to characterize the sediment samples to set limiting value on the acceptable equivalent dose recommended by ICRP [17] to 1.5 mSv/y. The value must be less than unity which corresponds to the upper limit of Ra_equ_ (370 Bq/kg) [15], [18], [19], [20]. The H_ext_ was calculated using Eq. (6)

(6) $$H_{ext}=\left\{\frac{C_{U}}{370}+\frac{C_{Th}}{259}+\frac{C_{K}}{4810}\right\}\leq 1$$

where, C_U_, C_Th_ and C_K_ are the average activity concentrations of U-238, Th-232 and K-40 in Bq/kg respectively. For the radiation hazard to be at the acceptable limit, it is recommended that the H_ext_ must be less than a unity. The estimated H_ex_ index for all the sediment samples ranges from 0.21 to 0.38 with a mean value of 0.31. The highest value of 0.38 was found in site 2, while the lowest value of 0.21reported in site 7. Comparing the mean value from this present study, it is lower than the recommended value of ≤ 1 according to [16]

#### Annual effective dose rate (AEDR)

To determine the AEDR, it is necessary to use the conversion coefficient of the absorbed dose in the air to the effective dose (0.7 Sv/Gy), and the outdoor occupancy factor (0.2 Sv/Gy) recommended by [16,17]. The AEDR is calculated using Eq. (7)

(7) $$AEDR=DC\times \;1.23\;\times 10^{-3}\;mSv/y$$

The AEDR from the measured samples is presented in Table 4 with the values range between 0.05 and 0.08 mSv/y. The mean value of 0.07 mSv/y was noted in the sediment samples of Iju river with the highest value of 0.08 mSv/y recorded in sites 2, 9, 12, and 13, respectively, while the lowest value reported in sites 5, 7, 8, and 10, respectively. In contrast, this present study with a mean value of 0.08 mSv/y annual effective dose surpass the world's average value of 0.07 mSv/y according to [16,17] by a factor 0.01.

#### Gamma activity index representations (I_ɣ_)

The estimation of the level of distribution of values of the gamma index in sediment samples are presented in Table 4. The gamma index is related to the annual dose rate attributed due to the excess external gamma radiation caused by Iju river sediments. The value of Iɣ ∠ 2 corresponds to a dose rate of the criterion of 0.30 mSvy-1, whereas 2 < Iɣ ∠ 6 corresponds to a criterion of 1 mSv/y whereas a gamma activity index ∠ 0.5 corresponds to 0.3 mSv/y if the materials are in a bulk quantity such as soil sediments [17,^22^,28]. If the Iɣ for sediment is greater than 6, such material should be avoided since it corresponds to dose rate higher than 1 mSv/y [17] which is presumed to be the highest dose rate value recommended for the general public [16]. The gamma index representation (Iy) is calculated using Equation (8) as suggested by [23,28]

(8) $$I\gamma =\frac{CRa}{300BqKg^{-1}}+\frac{CTh}{200BqKg^{-1}}+\frac{CK}{3000BqKg^{-1}}$$

The Iɣ for the measured sediment samples from Iju River varied between 0.30 and 0.55 with a mean value of 0.42. The highest value was noted in site 2 with a value of 0.55, while the lowest value of 0.30 reported in Site 7 as shown in Table 4. The mean value of 0.42 from the current study indicates that a dose rate delivered by the Iju river sediment samples is smaller than the annual effective dose constraints of 1 mSvy^−1^. As such, this sediments could be exempted from restrictions concerning radiological and radioactivity risks

#### Alpha index (Iα)

The Iα has been developed as an assessment of the excess alpha radiation exposure caused by the inhalation originating from building materials. The alpha index in this present study was calculated using Eq. (9) [24,25] is:

(9) $$I\alpha =\frac{CRa}{200BqKg^{-1}}$$

where C_Ra_ is the concentration of radium equivalent activity concentration in Bq/kg found in the measured sediment samples. If the radium activity level in Iju river sediment surpasses the values of 200 Bq/kg, there is tendency that the radon exhalation from the sediment may attribute to indoor radon concentrations exceeding 200 Bqm^−3^. The International Commission on Radiation Protection recommended a safe limit level of 200 Bqm^−3^ for radon exhalation to the general public (ICRP, [17,25]. At the same time, if this radium activity level is below 100 Bq/kg, it means that radon exhalation from the sediment sample may not attribute to the indoor concentration greater than 200 Bqm^−3^ [25,28]. The results of Iα from this study ranges from 0.40 to 0.7 with a mean value of 0.57 as shown in Table 4. This mean value is not up to the recommended exempted value and the recommended upper limit for radon concentrations which are 100 Bq/kg and 200 Bq/kg, respectively, in sediment materials [25,28]. It is noted that the upper limit of radon concentration (Iα) is equal to 1 [25]. The lower value indicates that the radon exhalation from all the sediment samples will attribute to indoor concentration lower than 200 Bq/kg.

#### Activity utilization index (AUI)

To determine the level of AUI from different combinations of the U-238, Th-232 and K-40 in the sediment samples of Iju river can be calculated using the following Equation 10:

(10) $$AUI=\left(\frac{C_{Ra}}{50\;\text{Bqkg}-1}\right)fRa+\left(\frac{C_{Th}}{50\;\text{Bqkg}-1}\right)fTh+\left(\frac{C_{K}}{500\;\text{Bqkg}-1}\right)fk$$

Where C_Th_, C_Ra_ and C_K_ are the actual values of the activities per unit mass (Bqkg^−1^) of U-238, Th-232 and K-40, respectively, in the assessed river sediments. fTh, fRa and fK are the fractional contributions of the total dose rate in air attributed to gamma radiation from the actual activity concentration from the measured radionuclides. The AUI is 2 by definition and is deemed to imply a dose rate of 80 nGyh^−1^ [16,^17^,25]. All the values are presented in Table 4. The AUI for all the measured samples varied from 0.46 to 0.89, with a mean value of 0.69. The highest value of 0.89 reported in site 13, whereas the lowest value of 0.46 was found in site 7. The mean value of 0.69 for the AUI satisfies the suggestion that the AUI must be less than 2, which corresponds to the annual effective dose of < 0.3 mSv/*y* ^−^ ^1^ according to [26]. This may not pose any radiation exposure to the people residing in the area.

#### Excess lifetime cancer risk (ELCR)

To estimate the probability of cancer risk to any population from the exposure to the radiation is determined by the Excess lifetime cancer risk (ELCR) which is calculated according to [27,28] using Equation 11.

(11) ELCR=AEDRXDLXRF

where AEDR, DL and RF are the annual effective dose equivalent, Duration of life (70 yrs) and risk factor (0.05 Sv^−1^), respectively. The risk factor is the fatal cancer risk per Sievert. For stochastic effect, the International Commission on the Radiological Protection (ICRP 60) uses a value of 0.05 for the general public [27]. The values from the sediment samples are presented in Table 4. The values vary between 0.16 and 0.30, with a mean value of 0.23. Comparing the mean value of 23 × 10^−2^ for ELCR with the average world value of 0.29 × 10^−3^ suggested by [16,^17^,29], this present study is higher by a factor of 0.03.

### Multivariate statistical analysis of relationship between U-238, Th-232 and K-40

The relationship between concentrations of U-238, Th-232, K-40 and sampling days, sampling sites were statistically estimated in all the sediment samples presented in Tables 5 to 10. Tables 8 to 10 show the ANOVA for 13 sites from the data taken within three different days with an alpha level of 0.05. The rows represent the variations between the stations, while the columns represent the variation with days. This analysis measures the asymmetry of the probability distribution of real value random variables which has many benefits to this current study. Since the data are asymmetrically distributed, the normal distribution has a skewness of zero, which perhaps, in reality, may not be perfectly symmetric according to [23]. For U-238 in Bq/Kg presented in Table 5, there is significant variation for both factors considered from the p-values observed. This signifies that some other factor(s) is/are not at play here, and the ones being considered are not the cause of the variations.

**Table 5:** ANOVA FOR U-238(Bq/Kg).

*Source of Variation*	*SS*	*df*	*MS*	*F*	*P-value*	*F crit*
Rows	117.3184	12	9.776536	0.363244	0.964499	2.18338
Columns	52.08335	2	26.04168	0.96757	0.394341	3.402826
Error	645.9482	24	26.91451			
Total	815.35	38				

In Table 5, both factors explain the variations properly. They both have p-values more than the alpha level of significance of 0.05.

**Dependent and Independent Variables for the Statistical Analysis**

U-238(Bq/Kg) = 25.92 - 2.01 * Day1 + 0.72 * Day2 + 1.16 * ST1 + 0.96 * ST2 - 2.54 * ST3 + 0.34 * ST4 - 0.28 * ST5 + 3.12 * ST6 - 3.16 * ST7 + 0.30 * ST8 + 0.96 * ST9 - 1.96 * ST10 + 2.34 * ST11 + 0.67 * ST12

Th- (Bq/Kg) = 73.43 - 20.34 * Day1 - 21.65 * Day2 - 26.0 * ST1 - 18.7 * ST2 - 21.1 * ST3 - 26.8 * ST4 - 29.4 * ST5 - 30.9 * ST6 - 22.2 * ST7 - 37.5 * ST8 - 13.5 * ST9 - 1.5 * ST10 - 0.2 * ST11 - 4.1 * ST12

K-(Bq/Kg) = 558 + 1.7 * Day1 - 75.9 * Day2 - 213 * ST1 + 61 * ST2 - 2 * ST3 - 76 * ST4 - 147 * ST5 + 49 * ST6 - 63 * ST7 - 67 * ST8 - 65 * ST9 - 95 * ST10 - 246 * ST11 + 104 * ST12

The dependent variables are U-238(Bq/Kg), Th- (Bq/Kg) and K-(Bq/Kg). The independent variables are Day1, Day2 and St1 – St 12 (station 1 – 12). Finding the estimated values for the dependent variables at any day and at any station will require replacing the Day and station (St) of interest with “1” and setting every other day and station to zero “0”.

**Regression Analysis:** U-238 in Bq/kg versus Day1, Day2, Day3, ST1, ST2, ST3, ST4, ST5, **…**

The following terms cannot be estimated and were removed: Day3, ST13

Method: Categorical predictor coding (1, 0)

In Table 5, the regression model is not significant. The model summary also shows low values.

Regression Equation

U-238(Bq/kg) = 25.92 + 0.0 Day1_0 - 2.01 Day1_1 + 0.0 Day2_0 + 0.72 Day2_1 + 0.0 ST1_0 + 1.16 ST1_1 + 0.0 ST2_0 + 0.96 ST2_1 + 0.0 ST3_0 - 2.54 ST3_1 + 0.0 ST4_0 + 0.34 ST4_1 + 0.0 ST5_0 - 0.28 ST5_1 + 0.0 ST6_0 + 3.12 ST6_1 + 0.0 ST7_0 - 3.16 ST7_1 + 0.0 ST8_0 + 0.30 ST8_1 + 0.0 ST9_0 + 0.96 ST9_1 + 0.0 ST10_0 - 1.96 ST10_1 + 0.0 ST11_0 + 2.34 ST11_1 + 0.0 ST12_0 + 0.67 ST12_1

In Table 6, both factors explain the variation properly. They both have p-values less than the alpha level of significance of 0.05

**Table 6:** The regression model for U-238 in Bq/kg Analysis of Variance.

Source	DF	Adj SS	Adj MS	F-Value	P-Value
Regression	14	169.402	12.1001	0.45	0.939
Day 1	1	26.253	26.2526	0.98	0.333
Day 2	1	3.384	3.3842	0.13	0.726
ST 1	1	2.027	2.0271	0.08	0.786
ST 2	1	1.373	1.3728	0.05	0.823
ST 3	1	9.696	9.6965	0.36	0.554
ST 4	1	0.173	0.1726	0.01	0.937
ST 5	1	0.116	0.1162	0.00	0.948
ST 6	1	14.582	14.5821	0.54	0.469
ST 7	1	14.978	14.9784	0.56	0.463
ST 8	1	0.134	0.1343	0.00	0.944
ST 9	1	1.373	1.3728	0.05	0.823
ST 10	1	5.760	5.7604	0.21	0.648
ST 11	1	8.184	8.1842	0.30	0.586
ST 12	1	0.678	0.6784	0.03	0.875
Error	24	645.948	26.9145		
Total	38	815.350			

**Regression Analysis:** Th-232 in Bq/kg versus Day1, Day2, Day3, ST1, ST2, ST3, ST4, ST5, **…**

The following terms cannot be estimated and were removed: Day3, ST13

Method: Categorical predictor coding (1, 0)

In Table 7, seven parameters are significant. The regression model itself is significant. The model summary has values that are higher than the previous models.

**Table 7:** ANOVA for Th-232 (Bq/Kg).

*Source of Variation*	*SS*	*df*	*MS*	*F*	*P-value*	*F crit*
Rows	5910.474	12	492.5395	2.621478	0.021453	2.18338
Columns	3831.784	2	1915.892	10.19709	0.000623	3.402826
Error	4509.27	24	187.8862			
Total	14,251.53	38				

Regression Equation

Th- (Bq/kg) = 73.43 + 0.0 Day1_0 - 20.34 Day1_1 + 0.0 Day2_0 - 21.65 Day2_1 + 0.0 ST1_0 - 26.0 ST1_1 + 0.0 ST2_0 - 18.7 ST2_1 + 0.0 ST3_0 - 21.1 ST3_1 + 0.0 ST4_0 - 26.8 ST4_1 + 0.0 ST5_0 - 29.4 ST5_1 + 0.0 ST6_0 - 30.9 ST6_1 + 0.0 ST7_0 - 22.2 ST7_1 + 0.0 ST8_0 - 37.5 ST8_1 + 0.0 ST9_0 - 13.5 ST9_1 + 0.0 ST10_0 - 1.5 ST10_1 + 0.0 ST11_0 - 0.2 ST11_1 + 0.0 ST12_0 - 4.1 ST12_1

In Table 8, the p-values are way beyond the level of significance. The variation is explained by other factors but not the two considered. They both have p-values greater than the alpha level of significance of 0.05.

**Table 8:** The regression model for Th-232 in Bq/kg Analysis of Variance.

Source	DF	Adj SS	Adj MS	F-Value	P-Value
Regression	14	9742.3	695.88	3.70	0.002
Day 1	1	2690.4	2690.36	14.32	0.001
Day 2	1	3046.3	3046.26	16.21	0.000
ST 1	1	1014.7	1014.73	5.40	0.029
ST 2	1	524.6	524.61	2.79	0.108
ST 3	1	665.9	665.9	3.54	0.072
ST 4	1	1079.1	1079.08	5.74	0.025
ST 5	1	1295.9	1295.89	6.90	0.015
ST 6	1	1433.6	1433.62	7.63	0.011
ST 7	1	736.1	736.09	3.92	0.059
ST 8	1	2107	2106.99	11.21	0.003
ST 9	1	274.4	274.38	1.46	0.239
ST 10	1	3.2	3.19	0.02	0.897
ST 11	1	0.1	0.09	0.00	0.982
ST 12	1	25.5	25.48	0.14	0.716
Error	24	4509.3	187.89		
Total	38	14,251.5			

**Regression Analysis: KBq/kg) versus Day1, Day2, Day3, ST1, ST2, ST3, ST4, ST5, …**

The following terms cannot be estimated and were removed: Day3, ST13

Method

Categorical predictor coding (1, 0)

Again, in Table 9, none of the parameters are significant.

**Table 9:** ANOVA for K-40 (Bq/Kg).

*Source of Variation*	*SS*	*df*	*MS*	*F*	*P-value*	*F crit*
Rows	382,199.3	12	31,849.94	0.785737	0.660025	2.18338
Columns	51,015.11	2	25,507.55	0.629271	0.541546	3.402826
Error	972,842.6	24	40,535.11			
Total	1,406,057	38

**Table 10:** The regression model for K-40 in Bq/kg Analysis of Variance.

Source	DF	Adj SS	Adj MS	F-Value	P-Value
Regression	14	433,214	30,943.9	0.76	0.695
Day 1	1	18	18.5	0.00	0.983
Day 2	1	37,412	37,412	0.92	0.346
ST 1	1	68,225	68,225.5	1.68	0.207
ST 2	1	5559	5558.5	0.14	0.714
ST 3	1	4	3.5	0.00	0.993
ST 4	1	8765	8765.1	0.22	0.646
ST 5	1	32,202	32,201.7	0.79	0.382
ST 6	1	3592	3591.6	0.09	0.769
ST 7	1	5961	5960.6	0.15	0.705
ST 8	1	6726	6725.6	0.17	0.687
ST 9	1	6382	6382.2	0.16	0.695
ST 10	1	13,550	13,550	0.33	0.569
ST 11	1	90,408	90,408.3	2.23	0.148
ST 12	1	16,289	16,289.4	0.40	0.532
Error	24	972,843	40,535.1		
Total	38	1,406,057			

Regression Equation

KBq/Kg) = 558 + 0.0 Day1_0 + 1.7 Day1_1 + 0.0 Day2_0 - 75.9 Day2_1 + 0.0 ST1_0 - 213 ST1_1 + 0.0 ST2_0 + 61 ST2_1 + 0.0 ST3_0 - 2 ST3_1 + 0.0 ST4_0 - 76 ST4_1 + 0.0 ST5_0 - 147 ST5_1 + 0.0 ST6_0 + 49 ST6_1 + 0.0 ST7_0 - 63 ST7_1 + 0.0 ST8_0 - 67 ST8_1 + 0.0 ST9_0 - 65 ST9_1 + 0.0 ST10_0 - 95 ST10_1 + 0.0 ST11_0 - 246 ST11_1 + 0.0 ST12_0 + 104 ST12_1

In summary, the U-238 in Bq/Kg and Th-232 in Bq/Kg shows that the days have a more significant effect on the dependent variables compared to the stations. This significant variation may be due to the mineral characteristic with particle size and migration of altitude in bottom sediments. In summary, the first and the third parameters (U-238 and K-40) represented with their initials in the ANOVA Tables shows that they are significant. The days have a significant effect on the observations of these two parameters (as seen in Table 3 and 8, respectively). The site variable only has an effect on the Th-232 parameter. The regression model for Th-232 has significance effect and is higher than the contributions if U-232 and K-40 respectively, with these values (*S* = 13.7072; R-Sq (adj.) = 49.90% and R-Sq (pred.) = 16.45%, while for U-238 and K-40 do not show any significant values for R-Sq (adj.) = 0.00 and R-Sq (pred.) = 0.00. The important radionuclide parameter controlling the process is often located in Th-232 and may attributed to the higher levels of the radiological risks such as the activity utilization index and excess lifetime cancer risk, which are higher than the world average values.

## Conclusion

The background radiation gamma dose rates measured along Iju river soil sediments has shown significant variations in distributions within the study area which ranges from 21.6 to 58.9 nGy/h. The Iso-dose map with the highest background gamma dose rate (hotspot) was identified in site 13. The entitre area with backgeound dose rates ranges from. The highest activity concentrations of 31.8 ± 1.2 Bq/Kg, 48.0 ± 1.9 Bq/Kg and 852.9 ± 14.7 Bq/K for U-238, Th-232 and K-40 were found in site 13, 11 and 2, respectively. The mean activity levels of U-238, Th-232 and K-40 are within the range of the world average value except the value of K-40, which is approximately 0.19 times higher than the international recommended level. The highest mean value for excess lifetime cancer risks was 0.23 (23 × 10^−2^) which is 0.03 times higher than 0.029 (0.29 × 10^−3^) world average value. The regression model indicates that Th-232 attributes more radioactivity impacts on the soil sediments than the contributions of U-232 and K-40, as such, presumed to be the radionuclide parameter controlling the depositional process in the region. This study may be used as a baseline data for spatial distribution of gamma dose rates and radioactivity monitoring of coastal river sediments in Southwest Nigeria and beyond.

## Declaration of Competing Interest

All the authors do not have any conflict of interest or financial interest that could jeopardize this present article

## Appendix Supplementary materials

Image, image 1

Image, image 2

Image, image 3
