# Supplementary figures and images for: Spatial distribution of gamma radiation dose rates from natural radionuclides and its radiological hazards in sediments along river Iju, Ogun state Nigeria
Source: MethodsX. 2020 Oct 7;7:101086. doi: 10.1016/j.mex.2020.101086 (PMC7581974; doi:10.1016/j.mex.2020.101086)

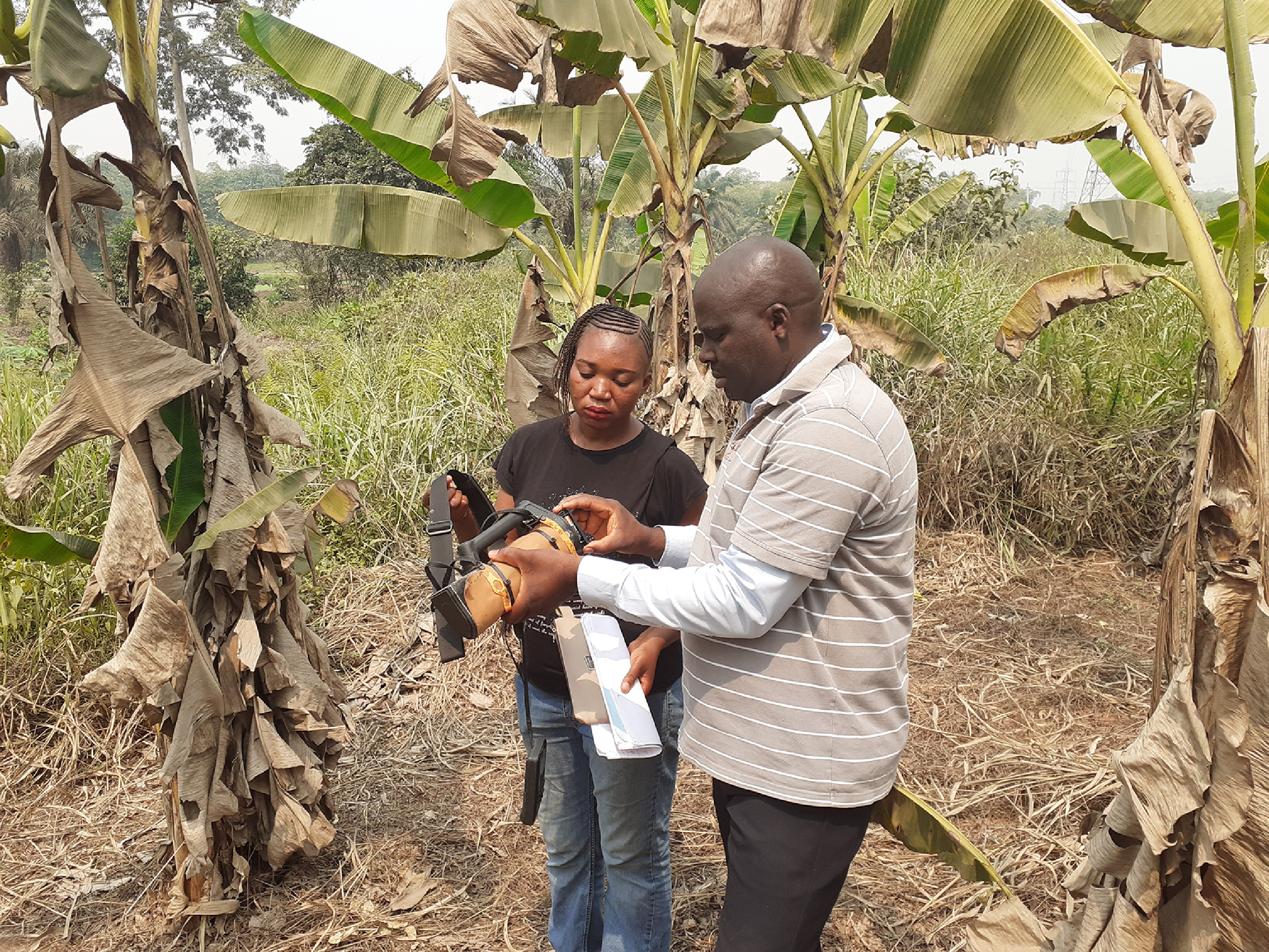

Supplement: Supplementary file 1 [file mmc1.jpg]

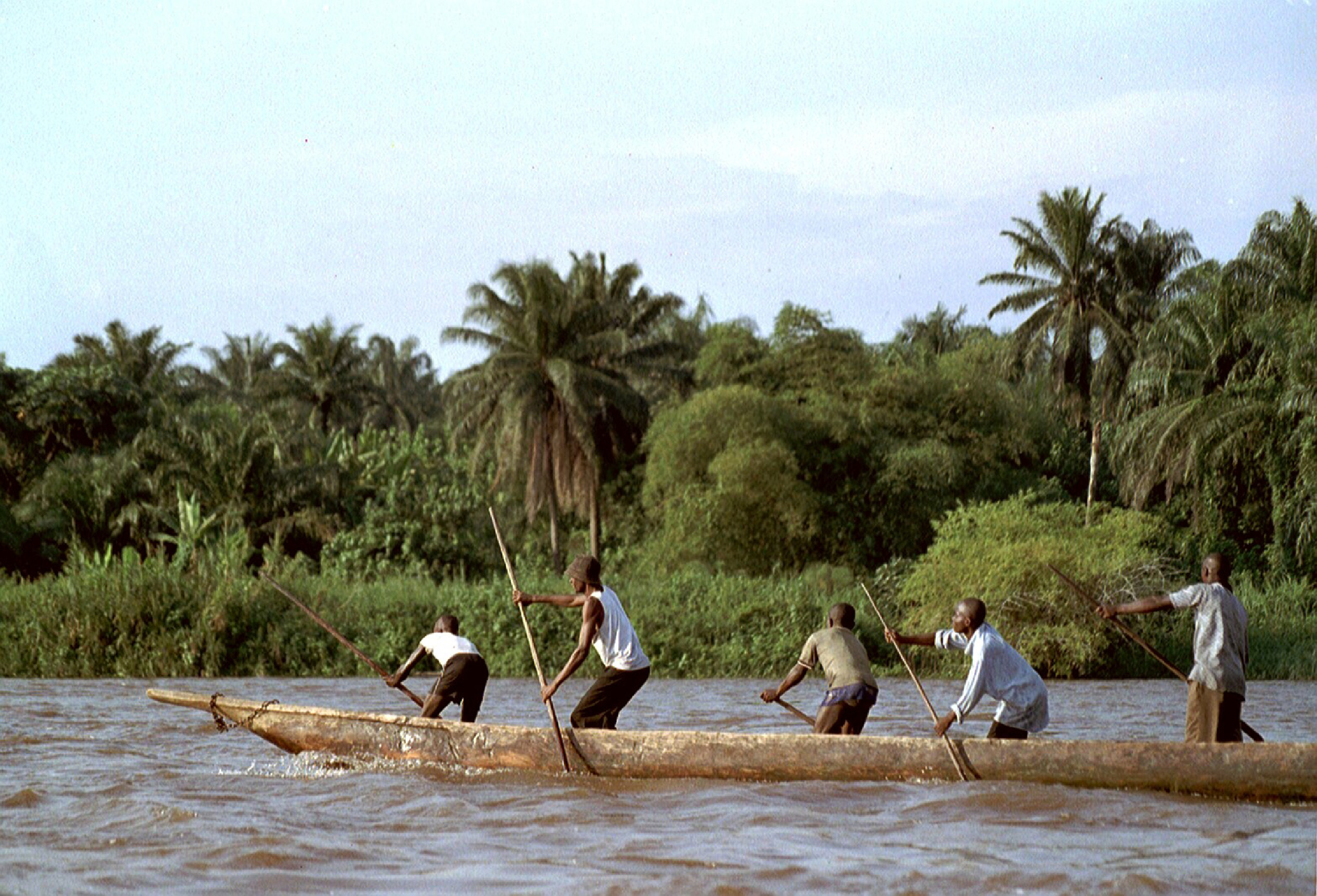

Supplement: Supplementary file 2 [file mmc2.jpg]

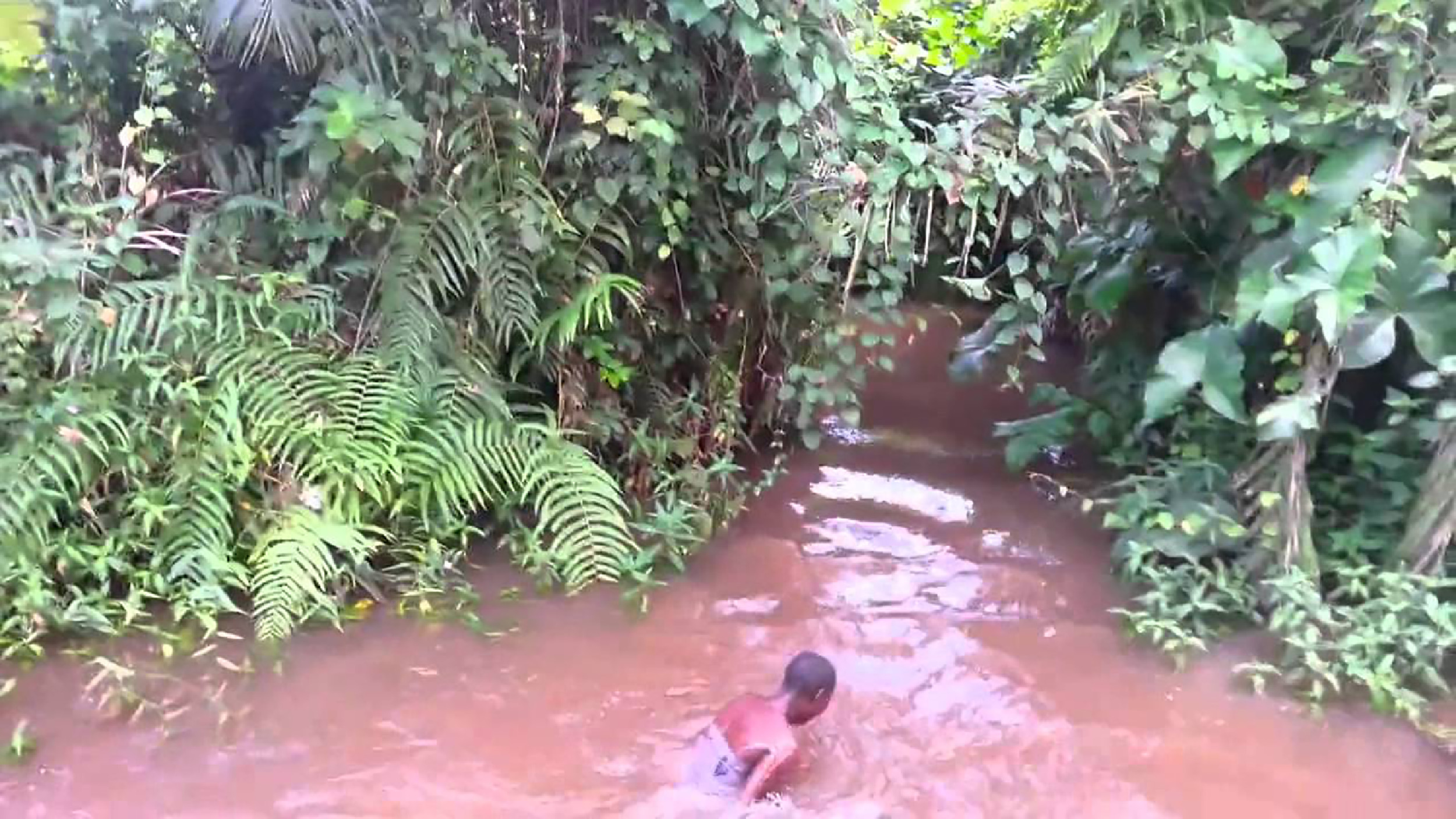

Supplement: Supplementary file 3 [file mmc3.jpg]
